# A glycoengineered anti-ROR1 antibody, GE-zilovertamab, selectively enhances antibody-dependent cellular cytotoxicity against chronic lymphocytic leukemia

**DOI:** 10.1093/abt/tbag001

**Published:** 2026-01-15

**Authors:** Md Kamrul Hasan, George Widhopf II, Thomas J Kipps

**Affiliations:** Center for Novel Therapeutics, Moores Cancer Center, University of California San Diego, 9310 Athena Circle, La Jolla, CA 92037-0809, United States; Center for Novel Therapeutics, Moores Cancer Center, University of California San Diego, 9310 Athena Circle, La Jolla, CA 92037-0809, United States; Center for Novel Therapeutics, Moores Cancer Center, University of California San Diego, 9310 Athena Circle, La Jolla, CA 92037-0809, United States

**Keywords:** CLL, antibody, ROR1, ADCC, NK cells

## Abstract

Receptor tyrosine kinase-like orphan receptor 1 (ROR1) is selectively expressed on chronic lymphocytic leukemia (CLL) B cells and certain cancers, but is absent from normal B cells and healthy adult tissues. GE-zilovertamab, an afucosylated anti-ROR1 IgG1 antibody, is engineered to increase FcγRIIIA binding and thereby enhance antibody-dependent cellular cytotoxicity (ADCC). Co-culture assays were performed using CLL cell lines (MEC1, MEC1-ROR1) and primary CLL cells with Jurkat-Lucia™ NFAT-CD16, NK, or peripheral blood mononuclear cell effectors. Treatments included the anti-CD20 mAb rituximab, anti-ROR1 mAbs (GE-zilovertamab, zilovertamab), and the endocytosis inhibitor prochlorperazine, and ADCC was quantified. GE-zilovertamab showed significantly higher ADCC than its parental antibody and activity that was comparable to that of rituximab. We find that the endocytosis inhibitor prochlorperazine further increased this effect. GE-zilovertamab is a promising next-generation immunotherapeutic for CLL, combining selective targeting of ROR1 with the potential to reduce therapy-induced immunodeficiency compared with anti-CD20 antibodies.

**Statement of Significance**

An afucosylated anti-receptor tyrosine kinase-like orphan receptor 1 antibody potently kills receptor tyrosine kinase-like orphan receptor 1-expressing leukemic B cells while sparing normal B cells, and this selective cytotoxicity is further enhanced by prochlorperazine. This combination has the potential to advance leukemia immunotherapy by targeting malignant cells while preserving humoral immunity, thereby addressing infection risks associated with anti-CD20 antibody regimens.

## Introduction

Chronic lymphocytic leukemia (CLL) is characterized by the gradual accumulation of monoclonal B lymphocytes in the blood, marrow, and lymphoid tissues and is frequently associated with acquired humoral immunodeficiency [1]. Current treatment strategies often employ anti-CD20 monoclonal antibodies (mAbs), such as rituximab or obinutuzumab, in combination with targeted agents including Bruton’s tyrosine kinase inhibitors (BTKi) or BCL-2 inhibitors (BCL2i), resulting in improved patient outcomes [2]. However, anti-CD20 therapy further depletes normal B cells and can exacerbate the already heightened risk of infection in CLL patients with underlying hypogammaglobulinemia [3]. This risk has become even more evident since the COVID-19 pandemic, in which preservation of humoral immunity is crucial for defense against infection, and literature reviews and meta-analyses report increased infection rates and reduced antibody responses in CLL patients treated with anti-CD20 mAbs [4–6].

By contrast, the receptor tyrosine kinase-like orphan receptor 1 (ROR1) is selectively expressed on CLL cells and certain solid tumors, but not on normal adult B cells or healthy tissues [7]. Quantitative immunofluorescence studies have demonstrated that leukemia cells from most patients express approximately 2,000–11,000 surface ROR1 molecules per cell [8]. Moreover, leukemia cells from patients with more aggressive disease and shorter overall survival typically express more than 5,800 surface ROR1 molecules per cell [8], which is comparable to the relatively low numbers of surface CD20 found on most CLL cells (less than approximately 15,000 molecules per cell) [9]. Together, these features suggest that ROR1 offers a unique and specific target for immunotherapy in CLL.

Antibody-dependent cellular cytotoxicity (ADCC) is a key mechanism of action for many therapeutic antibodies, including anti-CD20 mAbs. However, the humanized anti-ROR1 mAb advanced to clinical trials, zilovertamab (formerly cirmtuzumab) [8], was primarily designed to inhibit Wnt5a-mediated ROR1 signaling [7], and has relatively limited capacity to direct ADCC against ROR1-expressing cells [10]. Fc glycoengineering through afucosylation can enhance ADCC of some mAbs by increasing FcγRIIIA binding on natural killer (NK) cells [11]. To test whether afucosylation could similarly enhance the ADCC activity of zilovertamab, an afucosylated anti-ROR1 IgG1 antibody, GE-zilovertamab, was developed using approaches previously applied to other mAbs [12].

In this study, co-culture assays with CLL cell lines, primary patient samples, and either NK or peripheral blood mononuclear cell (PBMC) effectors were used to assess the activity of GE-zilovertamab relative to that of the anti-CD20 mAb rituximab. GE-zilovertamab consistently mediated stronger ADCC than zilovertamab while sparing normal B cells. Furthermore, combining GE-zilovertamab with the endocytosis inhibitor prochlorperazine, which inhibits dynamin-mediated receptor internalization, further increased ADCC [13], supporting a therapeutic strategy that may enhance tumor clearance in CLL while preserving immune competence.

## Materials and methods

### Cell lines and culture conditions

MEC1 cells derived from CLL were obtained from DSMZ and maintained in RPMI-1640 medium with 10% FBS and 1% penicillin–streptomycin at 37°C in 5% CO_2_. NK92 cells from ATCC were cultured in MyeloCult™ H5100 medium containing 12.6% horse serum, 1% penicillin–streptomycin-l-glutamine, and 100 IU/ml IL-2. NK92-CD16 cells were generated by transducing NK92 cells with a lentivirus vector encoding a high-affinity allele of CD16A (V176) with an additional amino acid change at position 197 of serine to proline (S197P) that removes the ADAM17-mediated proteolytic cleavage site (IDT).

### Human samples and primary CLL cell preparation

Buffy coat samples were obtained from the San Diego Blood Bank. Primary CLL cells were collected from patients at UCSD Moores Cancer Center. PBMCs were isolated via Ficoll-Paque density centrifugation and cryopreserved in 90% FBS and 10% DMSO.

### Jurkat-Lucia™ NFAT-CD16 ADCC reporter assay

MEC1, MEC1-ROR1 (stably transfected with ROR1), or primary CLL target cells were co-cultured with Jurkat-Lucia™ NFAT-CD16 effector cells (Invivogen) expressing FcγRIIIA (CD16A, V158, or F158 allotype) for 6 h at 37°C. Cells were treated with rituximab, GE-zilovertamab, or zilovertamab at various concentrations, with or without 50 nM prochlorperazine pretreatment for 1 h. Luminescence was measured as a surrogate for Fc receptor engagement.

## Results

### GE-zilovertamab enhances the ADCC efficacy induced by Jurkat-Lucia™ NFAT-CD16 effector cells

**Figure 1:**
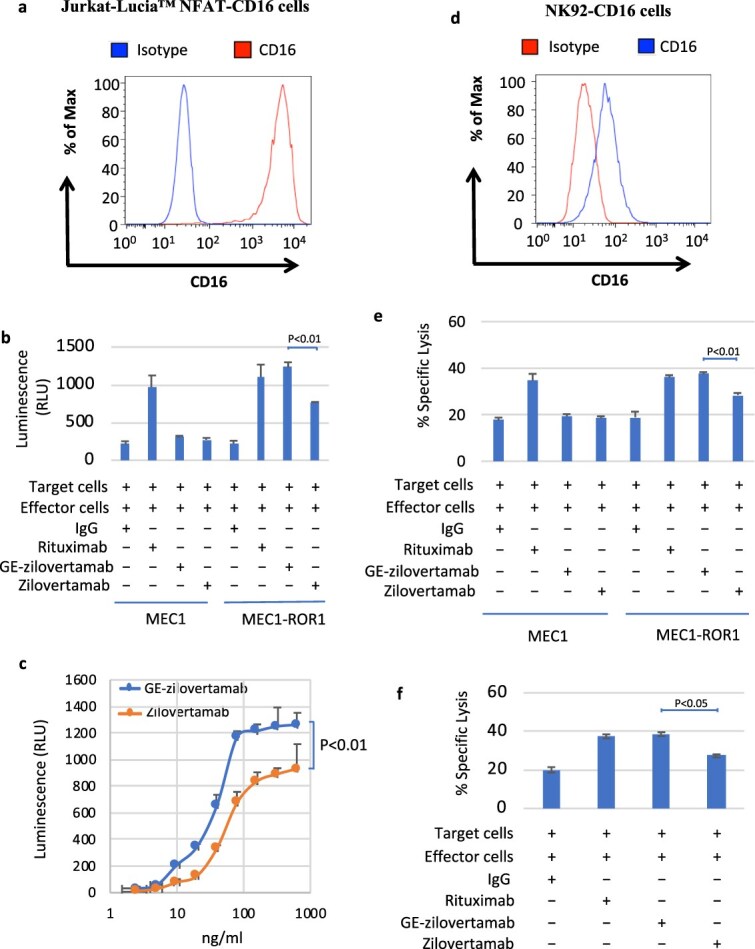
(a) Flow cytometric analysis of cell-surface high-affinity CD16 (FcγRIIIA; V158) expression on Jurkat-Lucia™ NFAT-CD16 cells. Fluorescence histograms show isotype control mAb (blue) and CD16-FITC (red). (b) Jurkat-Lucia™ NFAT-CD16 effector cells (e) were co-cultured with MEC1 or MEC1-ROR1 target cells (T) (E:T = 20:1; 6 h; 37°C) in the presence or absence of anti-CD20 (rituximab) or anti-ROR1 mAbs (GE-zilovertamab, zilovertamab; 100 ng/ml). Data are mean ± SD; unpaired two-tailed Student’s *t* test, *P* < .01. (c) Dose-dependent activation of CLL target cells (T) co-cultured with Jurkat-Lucia™ NFAT-CD16 effector cells (E:T = 20:1; 6 h; 37°C) and exposed to serial dilutions of anti-ROR1 mAbs (GE-zilovertamab, zilovertamab). Luminescence was measured after 6 h. data are mean ± SD; one-way ANOVA, *P* < .01. (d) Flow cytometric detection of CD16A on NK92-CD16 (FcγRIIIa; F176V/S197P mutated). Isotype control mAb (red) and CD16-FITC (blue) are shown. (e) ^51^Cr-labeled MEC1 or MEC1-ROR1 target cells (T) were co-cultured with NK92-CD16 effector cells (E:T = 2:1; 6 h; 37°C) with or without rituximab or anti-ROR1 mAbs (GE-zilovertamab, zilovertamab; 100 ng/ml). Percent lysis was calculated from ^51^Cr release relative to TCA-treated cells. Data are mean ± SD; two-tailed Student’s *t* test, *P* < .01. (f) ^51^Cr-labeled primary CLL cells (T) were co-cultured with NK92-CD16 effector cells (E:T = 10:1; 6 h; 37°C) with or without rituximab or anti-ROR1 mAbs (GE-zilovertamab, zilovertamab; 100 ng/ml). Percent specific lysis was calculated as in panel e. Data are mean ± SD; two-tailed Student’s *t* test, *P* < .05.

Glycan analysis showed that GE-zilovertamab is effectively afucosylated in the Fc region (Supplemental Table S1). Cytotoxicity was evaluated using the human CLL cell line MEC1, which endogenously expresses CD20 but not ROR1, MEC1 cells stably transfected to express ROR1 (MEC1-ROR1) (Supplemental Fig. S1a and b), and primary CLL B cells expressing both ROR1 and CD20 (Supplemental Fig. S1c and d). These target cells were co-cultured with Jurkat-Lucia™ NFAT-CD16 effector cells stably expressing the high-affinity CD16A (FcγRIIIA; V158 allotype) (Fig. 1a) at an effector-to-target (E:T) ratio of 20:1 for 6 h at 37°C in the presence or absence of the anti-CD20 mAb rituximab or anti-ROR1 mAbs GE-zilovertamab or zilovertamab. Luminescence was then measured as a readout of Fc receptor engagement. MEC1 cells demonstrated CD20-dependent ADCC with rituximab but not with GE-zilovertamab or zilovertamab (Fig. 1b). In contrast, MEC1-ROR1 cells showed increased luminescence and ADCC-mediated killing with rituximab, GE-zilovertamab, or zilovertamab compared with IgG-treated controls (Fig. 1b). Co-culture studies using MEC1-ROR1 targets and Jurkat-Lucia™ NFAT-CD16 effectors expressing either high-affinity CD16A (FcγRIIIA; V158 allotype) or low-affinity CD16A (FcγRIIIA; F158 allotype) showed that GE-zilovertamab enhanced ADCC relative to zilovertamab in both effector cell populations (Supplemental Fig. S2a and b). Similarly, primary CLL cells responded to all three antibodies, with higher luminescence for GE-zilovertamab than for zilovertamab, and ADCC dose–response curves revealed that GE-zilovertamab was more potent than its parental antibody (Fig. 1c).

### GE-zilovertamab enhances ADCC-mediated by NK92-CD16 effector cells

Chromium-51 release assays were performed using NK92-CD16 cells, a human NK line stably expressing high-affinity FcγRIIIA (Fig. 1d) [14]. Target cells (MEC1, MEC1-ROR1; Fig. 1e) or primary CLL cells (Fig. 1f) were labeled with ^51^Cr and co-cultured for 6 h at 37°C with NK92-CD16 effectors in the presence or absence of rituximab, GE-zilovertamab, or zilovertamab. Percent specific lysis was calculated relative to trichloroacetic acid (TCA)–treated cells. MEC1 cells, which lack ROR1, demonstrated ADCC only with rituximab, whereas MEC1-ROR1 and primary CLL cells (ROR1-positive) showed increased cytotoxicity with all three antibodies. GE-zilovertamab consistently produced greater lysis than zilovertamab, supporting its enhanced ADCC activity.

### GE-zilovertamab enhances ADCC-mediated by peripheral blood mononuclear cells

**Figure 2:**
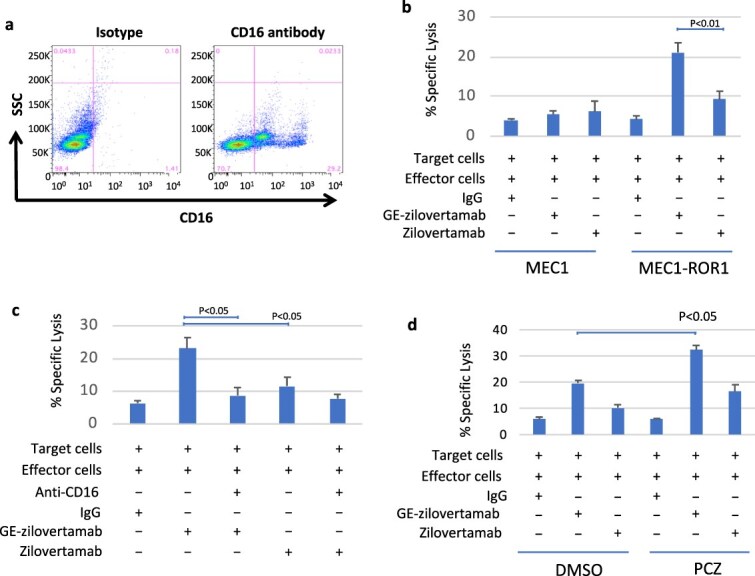
(a) Flow cytometric analysis of CD16 expression in peripheral blood mononuclear cells (PBMCs). Isotype control and CD16-FITC staining are shown. (b) ^51^Cr-labeled MEC1 or MEC1-ROR1 target cells (T) were co-cultured with PBMCs (E:T = 20:1; 6 h; 37°C; ≥25% CD16^+^) with or without anti-ROR1 mAbs (GE-zilovertamab, zilovertamab; 100 ng/ml). Percent lysis was calculated relative to TCA-treated controls. Data are mean ± SD; two-tailed Student’s *t* test, *P* < .01. (c) ^51^Cr-labeled primary CLL cells (T) were co-cultured with PBMCs (E:T = 50:1; 6 h; 37°C; ≥25% CD16^+^) with or without anti-CD16 or anti-ROR1 mAbs (GE-zilovertamab, zilovertamab; 100 ng/ml). Percent specific lysis was calculated. Data are mean ± SD; two-tailed Student’s *t* test, *P* < .05. (d) ^51^Cr-labeled MEC1-ROR1 cells were pre-treated for 1 h with DMSO or prochlorperazine (PCZ; 50 nM), then co-cultured with PBMCs (E:T = 20:1; 6 h; 37°C) with or without rituximab or anti-ROR1 mAbs (GE-zilovertamab, zilovertamab; 100 ng/ml). Percent lysis was calculated as in panel b. Data are mean ± SD; two-tailed Student’s *t* test, *P* < .01.

Flow cytometric analysis revealed that more than 25% of PBMCs from a healthy donor expressed CD16 (Fig. 2a). ^51^Cr-release assays were then performed by co-culturing ^51^Cr-labeled target cells (MEC1, MEC1-ROR1; Fig. 2b, or primary CLL cells; Fig. 2c) with PBMC effectors for 6 h at 37°C in the presence or absence of anti-ROR1 antibodies. MEC1 cells (ROR1-negative) did not exhibit ROR1-mediated ADCC. In contrast, MEC1-ROR1 and primary CLL cells (ROR1-positive) showed higher cytotoxicity with GE-zilovertamab or zilovertamab than with control IgG. Pretreatment of primary CLL cells with an anti-CD16 antibody inhibited ADCC, indicating a CD16-dependent mechanism (Fig. 2c). Across all PBMC experiments, GE-zilovertamab induced stronger cytotoxicity than zilovertamab.

### Endocytosis inhibitor prochlorperazine enhances ADCC-mediated killing

Cell viability assays showed that treatment of MEC1-ROR1 cells with prochlorperazine at 50–100 nM for 7 h did not reduce viability (Supplemental Fig. S3). On this basis, 50 nM prochlorperazine was used in subsequent experiments. Antibody-induced internalization of ROR1 was confirmed using zilovertamab conjugated with pH-sensitive pHrodo dyes, and prochlorperazine pretreatment inhibited this internalization (Supplemental Fig. S4). MEC1-ROR1 cells were then treated with DMSO or prochlorperazine for 1 h prior to co-culture with PBMCs and subsequently exposed for 6 h at 37°C to anti-ROR1 antibodies (GE-zilovertamab or zilovertamab; Fig. 2d). GE-zilovertamab again induced greater cytotoxicity than zilovertamab, and prochlorperazine pretreatment further increased ADCC-mediated killing with both antibodies, most notably with GE-zilovertamab. These findings support a model in which antibody binding promotes ROR1 internalization that can be limited by prochlorperazine, consistent with dynamin-dependent, clathrin-mediated endocytosis described for other receptors such as the epidermal growth factor receptor (EGFR) [15].

## Discussion

These studies demonstrate that GE-zilovertamab enhances ADCC activity beyond its parental antibody, zilovertamab, consistent with improved engagement of FcγRIIIA (CD16) by the glycoengineered Fc region. Both reporter and ^51^Cr-release assays, performed with engineered cell lines and primary CLL patient samples, support the superior functionality and ROR1-specific cytotoxicity of GE-zilovertamab. In PBMC co-cultures, blocking CD16 abrogated the cytotoxic response, confirming a CD16-dependent mechanism.

Importantly, the endocytosis inhibitor prochlorperazine further boosted ADCC, particularly with GE-zilovertamab. The data show that ROR1 undergoes antibody-induced internalization that can be inhibited by prochlorperazine, in a pattern consistent with dynamin-dependent, clathrin-mediated endocytosis described for EGFR [15]. ADCC directed by GE-zilovertamab against ROR1-expressing targets can be enhanced by prochlorperazine at concentrations that are readily achieved in vivo within dose ranges that are well tolerated and approved by regulatory agencies. Thus, prochlorperazine holds promise for repurposing as a combination partner to maximize the ADCC efficacy of GE-zilovertamab in patients with ROR1-positive malignancies.

ROR1 is a compelling therapeutic target in CLL and related malignancies, contributing to cancer cell proliferation and migration through diverse signaling pathways [7]. Zilovertamab has shown specificity and tolerability in early clinical studies [8], and the present work indicates that its glycoengineered variant, GE-zilovertamab, can offer improved effector function without loss of target selectivity. These data support a combined strategy of Fc glycoengineering and pharmacologic endocytosis inhibition as a rational approach to immunotherapy of ROR1-positive cancers.

This strategy is highly relevant to CLL therapy, where anti-CD20 mAbs (rituximab, obinutuzumab) used with targeted agents can improve patient outcomes but also contribute to immunodeficiency [16, 17]. Despite gains in progression-free survival, anti-CD20–containing regimens can increase the risk of opportunistic infections, exacerbate hypogammaglobulinemia, and impair vaccine responses [4–6]. Moreover, recent data show that anti-CD20–based therapies can raise rates of grade ≥ 3 infection and lead to prolonged reductions in immunoglobulin levels [18]. These risks are particularly concerning today for CLL patients with prior COVID-19 infection or poor vaccine responses [19]. In contrast, because ROR1 is not expressed on normal B cells [7], GE-zilovertamab has the potential to selectively target tumor cells while better preserving humoral immunity and minimizing infection risk. Monitoring immunoglobulin levels and infection rates should therefore be emphasized in future clinical trials of GE-zilovertamab, alone, or in combination with prochlorperazine.

An additional consideration is the relationship of GE-zilovertamab to ROR1-directed antibody–drug conjugates (ADCs) based on the same parental antibody [20]. ADCs and ADCC-optimized antibodies rely on distinct mechanisms and toxicity profiles: ADCs deliver cytotoxic payloads following internalization, whereas afucosylated antibodies such as GE-zilovertamab primarily mediate immune-effector–driven killing. A “naked” afucosylated antibody may therefore provide an alternative option for patients who cannot tolerate ADC-related toxicities or for combination regimens in which minimizing systemic exposure to cytotoxic payloads is desirable.

In summary, GE-zilovertamab, an afucosylated anti-ROR1 antibody, enhances ADCC compared with its parental antibody, likely through improved FcγRIIIA engagement. Prochlorperazine further augments cytotoxicity by limiting antibody-induced internalization of ROR1 and thereby stabilizing the target at the cell surface. While these findings are robust in vitro, future studies will be essential to define FcγRIIIA binding quantitatively, evaluate in vivo efficacy and safety, and determine the clinical benefit of GE-zilovertamab, alone and in combination with prochlorperazine, for patients with CLL and other ROR1-positive malignancies.

## Supplementary Material

Supplemental_material_tbag001

## Data Availability

The data that support the findings of this study are available upon reasonable request.

## Animal research statement

None declared.
