# Supplementary material for: A glycoengineered anti-ROR1 antibody, GE-zilovertamab, selectively enhances antibody-dependent cellular cytotoxicity against chronic lymphocytic leukemia
Source: Antib Ther. 2026 Jan 15;9(1):70–5. doi: 10.1093/abt/tbag001 (PMC12967326; doi:10.1093/abt/tbag001)
Supplement: Supplemental_material_tbag001 [file supplemental_material_tbag001.pdf]

Supplemental material

A Glycoengineered Anti-ROR1 Antibody, GE-zilovertamab, Selectively Enhances Antibody-Dependent Cellular Cytotoxicity Against Chronic Lymphocytic Leukemia

Supplemental materials and methods

GE-zilovertamab generation

The anti-ROR1 mAb GE-zilovertamab was generated by Evitria (Zurich, Switzerland). This afucosylated antibody was produced in Chinese hamster ovary (CHO) cells using Glymax technology.

Glycan analysis

We performed glycan analysis of GE-zilovertamab by liquid chromatography–mass spectrometry (LC-MS). The IgG antibody was digested with FabRICATOR protease in PBS supplemented with DTT. This protease cleaves IgG antibodies at a single site below the hinge which, together with reduction of intermolecular disulfide bonds by DTT, yields three subunits (scFc, LC, and Fd'). These subunits were analyzed by reverse-phase LC-MS on a Waters BioAccord LC-MS system equipped with a Waters BioResolve RP mAb column (2.1 × 50 mm). The amount of fucose was determined by calculating the average fucose content within the sugar chain at Asn297, relative to the sum of all glycostructures attached to Asn297. Asn297 may also be located approximately ±3 amino acids upstream or downstream (positions 294–300) due to minor sequence variations in antibodies. The Fc glycosylation profile of recombinantly expressed IgGs, including most approved therapeutic mAbs, is generally dominated by G0F and G1F structures, with lower amounts of G2F and afucosylated G0. In contrast, glycosylation analysis of GE-zilovertamab indicates that this antibody contains no fucose at the G0 or G1 structures (Table S1).

Table S1. Glycosylation Profile

<table>
<tr><th>Subunit</th><th>Glycoform</th><th>Percent</th><th>Afucosylation</th><th>High-mannose</th></tr>
<tr><td>scFc</td><td>Glycosylation</td><td>78.6</td><td rowspan="2">100</td><td rowspan="2">0.0</td></tr>
<tr><td>scFc</td><td>Glycosylation</td><td>21.4</td></tr>
</table>

Chromium-51 release assays with NK92-CD16

NK92 cells were stably transduced with a high-affinity, non-cleavable FcγRIIIA (V176, P197 mutations) to generate NK92-CD16 cells. MEC1, MEC1-ROR1, or primary CLL cells were labeled with chromium-51 (^{51}Cr) and co-cultured with NK92-CD16 cells at defined effector-to-target (E:T) ratios, with or without antibodies and with or without prochlorperazine, for 6 hours. Percent lysis was calculated relative to maximal ^{51}Cr release from TCA-treated controls.

PBMC-mediated ^{51}Cr-release assays

Thawed PBMCs (with >25% CD16-positive cells) were incubated with ^{51}Cr-labeled target cells at specified E:T ratios and treated with antibodies. In selected conditions, an anti-CD16 blocking antibody was added to assess FcγRIIIA dependence.

Prochlorperazine dose-finding and viability assay

MEC1-ROR1 cells were exposed to 10 nM–50 µM prochlorperazine for 7 hours, and viability was assessed using the Cell Counting Kit-8. Concentrations of 50–100 nM, which maintained cell viability, were used in subsequent functional assays.

Statistical analysis

Data are expressed as mean ± SD from at least three independent experiments. Two-tailed unpaired Student's t tests were used for two-group comparisons, and two-way ANOVA with Tukey's HSD post hoc testing was used for multiple-group comparisons. A p value < 0.05 was considered statistically significant. Analyses were performed using GraphPad Prism 6.0.

Figure S1

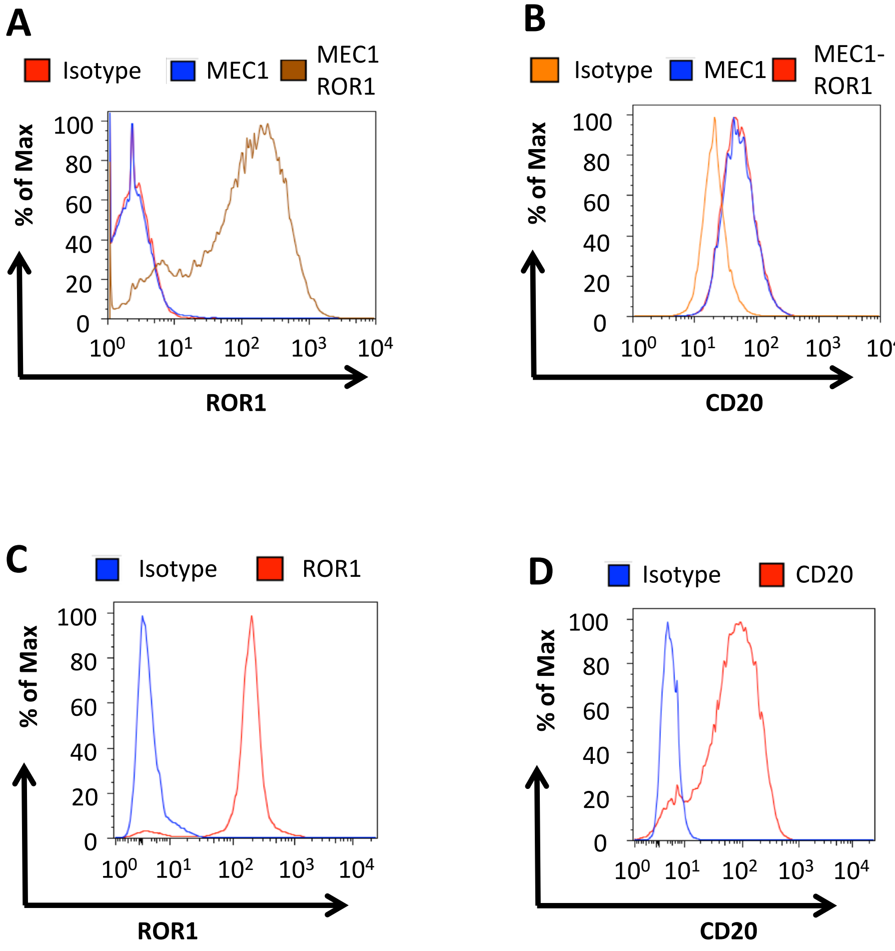

Figure S1. (A) Flow cytometry of MEC1 (blue) and MEC1-ROR1 (gold) cells stained with isotype control mAb (red) or 4A5-Alexa-647. (B) MEC1 (blue) and MEC1-ROR1 (red) cells stained with isotype control mAb (orange) or CD20-FITC. (C) Primary CLL cells stained with isotype control mAb (blue) or 4A5-Alexa-647 (red). (D) Primary CLL cells stained with isotype control mAb (blue) or CD20-FITC (red).

Figure S2

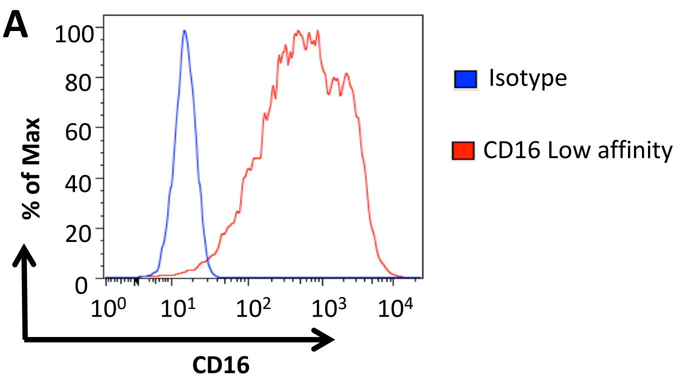

Figure S3. (A) Cell surface staining of Jurkat-Lucia™ NFAT-CD16 effector cells stably expressing the cell surface Fc receptor CD16-Low (FcγRIIIA; F158 allotype). Fluorescence of the cells after staining with a fluorochrome-labeled isotype control mAb (blue histogram) or CD16-FITC (red histogram).

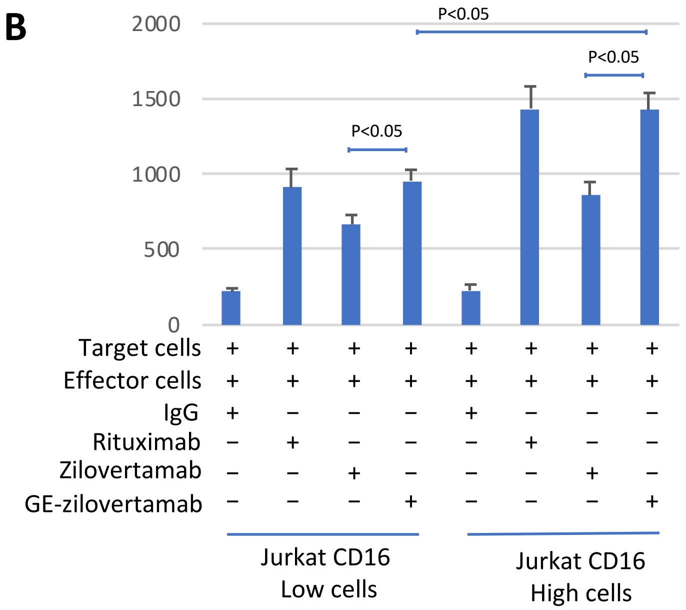

Figure S3. (B) MEC1-ROR1 target cells (T) were co-cultured with Jurkat-Lucia™ NFAT-CD16 effector cells (E) stably expressing the cell surface Fc receptor CD16A-Low (FcγRIIIA; F158 allotype) or CD16A-High (FcγRIIIA; V158 allotype) at E/T ratios = 20:1 for 6 hours at 37°C IgG or anti-CD20 mAb (rituximab) or anti-ROR1 mAbs such as zilovertamab or GE-zilovertamab at a concentration = 100 ng/ml. Data are shown as mean ± SD, 2-tailed Student's *t* test; $p < 0.05$.

Figure S3

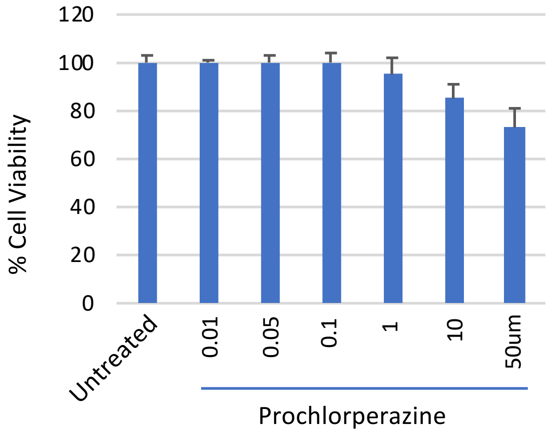

Figure S3. MEC1-ROR1 cells treated with prochlorperazine (various concentrations; 7 h) were assayed for viability. Percent viability normalized to untreated control. Data: mean ± SD (n=3).

Figure S4

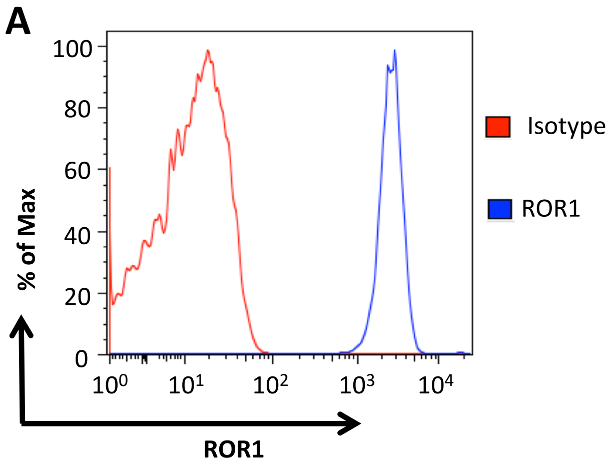

Figure S4. (A) Flow cytometry of JeKo-1 cells stained with isotype control mAb (red) or zilovertamab-Alexa-647 (blue).

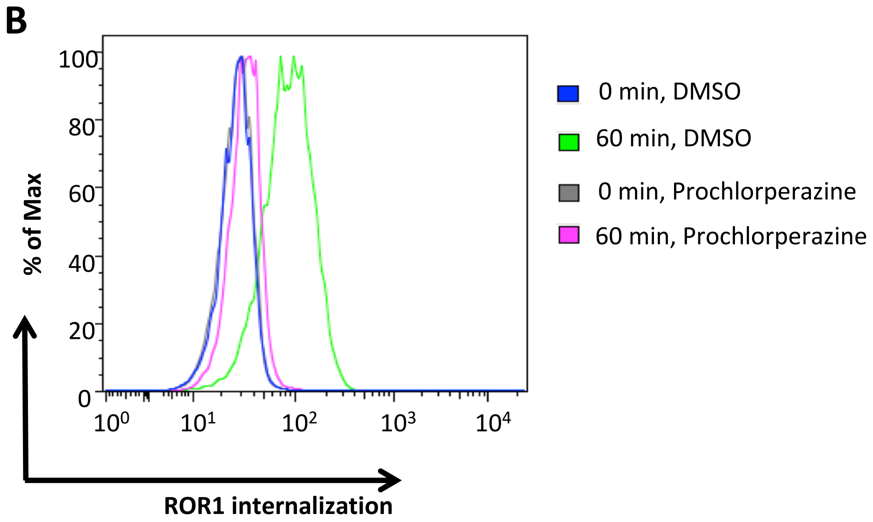

Figure S4. (B) Flow cytometry of JeKo-1 cells that were pre-treated 1 h with control solvent DMSO or prochlorperazine (50 nM), and stained with anti-ROR1 antibody zilovertamab conjugated with pH sensitive Rodo dye (Thermo fisher scientific, Waltham, MA, USA) at 37 ℃ after incubation at 0 minute (DMSO: blue, prochlorperazine : gray), or 60 minutes (DMSO: green, prochlorperazine: pink).
